# Peer Review of “Improved Alzheimer Disease Diagnosis With a Machine Learning Approach and Neuroimaging: Case Study Development”

**DOI:** 10.2196/73768

**Published:** 2025-04-21

**Authors:** Anonymous

**Keywords:** Alzheimer disease, computer-aided diagnosis system, machine learning, principal component analysis, linear discriminant analysis, t-distributed stochastic neighbor embedding, feedforward neural network, vision transformer architecture, support vector machines, magnetic resonance imaging, positron emission tomography imaging, Open Access Series of Imaging Studies, Alzheimer's Disease Neuroimaging Initiative, OASIS, ADNI

*This is a peer-review report for “Improved Alzheimer Disease Diagnosis With a Machine Learning Approach and Neuroimaging: Case Study Development.”*

## Round 1 Review

### General Comments

This paper [1] proposes a computer-aided diagnosis (CAD) system for Alzheimer disease (AD) using principal component analysis (PCA) and machine learning–based approaches. The authors claim that their system, which combines PCA for feature extraction with support vector machines (SVMs) and artificial neural networks (ANNs) for classification, achieves good accuracy in detecting AD from magnetic resonance imaging (MRI) and positron emission tomography (PET) images. However, the paper could be strengthened by addressing several areas for improvement.

### Specific Comments

#### Major Comments

1. Consideration of alternative methodologies: While the use of PCA, SVMs, and ANNs for AD classification is a valid approach, the authors should consider exploring more recent deep learning architectures, such as vision transformers, which have demonstrated state-of-the-art performance in medical image analysis. This would help to situate the work within the broader context of current research in the field.
2. Limited evaluation: The evaluation is limited to the Open Access Series of Imaging Studies (OASIS) dataset, which may not be representative of the diverse AD population. The authors should evaluate their system on larger and more diverse datasets, such as the Alzheimer’s Disease Neuroimaging Initiative (ADNI) dataset, to demonstrate its generalizability.

#### Minor Comments

1. Insufficient implementation details: The implementation details of the SVMs and ANNs are insufficient. The authors should specify the hyperparameters used, such as the kernel type and regularization parameters for SVMs, and the number of layers and neurons for ANNs.
2. Limited discussion: The discussion of the results is limited. The authors should provide a more in-depth analysis of the performance of their system, comparing it with other state-of-the-art methods and discussing the limitations and potential future directions.
3. The authors should ensure consistent formatting throughout the paper, including the use of italics for variables and proper capitalization in section headings.
4. The paper could be improved by using more precise language. For instance, instead of “good accuracy,” the authors could specify the exact accuracy percentage achieved by their system.

## Round 2 Review

### General Comments

This paper investigates the performance of various machine learning models in the diagnosis of AD using neuroimaging data. The authors propose a CAD system that uses PCA for feature extraction and SVMs, feedforward neural networks, and vision transformers for classification. The models are trained and evaluated on two datasets, OASIS and ADNI.

### Specific Comments

#### Major Comments

1. The paper claims that the proposed CAD system is effective in classifying patients with AD and healthy controls with high accuracy. However, the reported accuracies of 91.9% for OASIS and 88.6% for ADNI using PCA/SVM are not significantly higher than those achieved by existing state-of-the-art methods (eg, Li Y, Chen G, Wang G, et al. Dominating Alzheimer’s disease diagnosis with deep learning on sMRI and DTI-MD. *Front Neurol*. Aug 15, 2024;15:1444795. [doi: 10.3389/fneur.2024.1444795] [PMID: 39211812]). A more comprehensive literature review and comparison are needed to support the claim of the proposed system’s superiority.
2. The ADNI dataset includes not only patients with AD and healthy controls but also individuals with mild cognitive impairment (MCI). The paper does not explicitly mention whether MCI cases are included in the ADNI dataset used in this study and if patients with MCI are excluded. What is the reason?
3. The paper’s conclusion that the “PCA/SVM scheme is much better at predicting AD than the other models” is not supported by the results presented. The vision transformer model with data augmentation consistently outperforms PCA/SVM in terms of accuracy and other metrics. There are no obvious reasons data augmentation is unwanted either.

#### Minor Comments

1. The paper claims to use a multimodal system, combining both MRI and PET images. However, it does not compare the multimodal system’s performance against single-modal systems using only MRI or PET images. Such a comparison would help to rationalize the conclusion that the multimodal system truly improves upon single-modal systems.

## Abbreviations

AD: Alzheimer disease
ADNI: Alzheimer’s Disease Neuroimaging Initiative
ANN: artificial neural network
CAD: computer-aided diagnosis
MCI: mild cognitive impairment
MRI: magnetic resonance imaging
OASIS: Open Access Series of Imaging Studies
PCA: principal component analysis
PET: positron emission tomography
SVM: support vector machine
